# Characteristics of Patients With Advanced or Metastatic Urothelial Cancer Unable to Complete Six Cycles Chemotherapy

**DOI:** 10.1002/cam4.71873

**Published:** 2026-06-19

**Authors:** Francesca Jackson‐Spence, Domiziana Aspden, Vishwani Chauhan, Sofia Diaz, Catherine Graham, Matthew Young, Elizabeth Nally, John Connor Wells, Sara Coca Membribes, Charlotte Ackerman, Bernadett Szabados, Thomas Powles

**Affiliations:** ^1^ Barts Cancer Institute Queen Mary University of London London UK; ^2^ Department of Medical Sciences University of Turin Torino Italy; ^3^ Department of Medical Oncology Barts Health NHS Trust London UK; ^4^ University Hospital A Coruña A Coruña Spain; ^5^ Centre for Experimental Cancer Medicine Queen Mary University of London London UK

**Keywords:** avelumab maintenance, bladder cancer, chemotherapy cycles, metastatic urothelial cancer

## Abstract

**Background:**

The optimal number of chemotherapy cycles prior to maintenance avelumab in frontline metastatic urothelial cancer (mUC) remains unknown. We hypothesize that baseline characteristics can identify which patients will complete six cycles of chemotherapy without radiographic disease progression, versus those who may benefit from stopping chemotherapy earlier and switching to maintenance avelumab.

**Methods:**

A retrospective audit was performed at Barts Cancer Centre for consecutive patients from January 2010 until August 2023. Patients who received frontline chemotherapy for mUC were included. Patients who completed six cycles of chemotherapy without radiographic disease progression (PD) were compared to those who discontinued treatment earlier or progressed during chemotherapy. Baseline characteristics and treatment factors were compared between patient groups using chi‐squared tests (SPSS v29).

**Results:**

194 patients receiving frontline platinum‐based chemotherapy were identified. Only 85 of 193 patients (44%) completed six cycles without progression. Baseline characteristics were broadly comparable between patients who completed six cycles and those who did not, including ECOG performance status 0–1 (84% vs. 76%), presence of liver metastases (17% vs. 22%), baseline hemoglobin < 100 g/L (6% vs. 19%), and cisplatin use (59% vs. 54%), respectively. Early dose reduction (DR) prior to cycle 4 was the only factor significantly associated with failure to complete six cycles of chemotherapy without PD (14% vs. 43%, *p* < 0.001). Among patients requiring early dose reduction, only 19% subsequently completed six cycles without progression, while 43% progressed before reaching cycle 6, with a median PFS of 3 months (95% CI: 2.4–4.1).

**Conclusion:**

Baseline characteristics do not reliably predict the ability to complete six cycles frontline chemotherapy in mUC. However, early dose reduction in the first four cycles highly predicted inability to complete six cycles of chemotherapy.

## 1 Background

Although the Antibody Drug Conjugate (ADC) and Immune checkpoint inhibitor (IO) combination of Enfortumab Vedotin and Pembrolizumab (EVP) has become the standard of care for first‐line treatment of metastatic urothelial carcinoma (mUC) [1], platinum‐based chemotherapy followed by avelumab maintenance remains an important treatment strategy where this regime is unavailable [2]. The eligibility criteria to receive maintenance avelumab requires a patient to receive between four and six cycles of platinum‐based chemotherapy, without radiographic disease progression. Maintenance immune therapy should start within 10 weeks of the final chemotherapy dose [3].

Despite its widespread use, the optimal duration of frontline chemotherapy for mUC remains unclear. Planning for six cycles is globally accepted standard practice; however, prospective data addressing chemotherapy duration in the maintenance setting are emerging. The phase II DISCUS trial randomized patients to receive either three or six cycles of platinum‐based chemotherapy, followed by avelumab maintenance. Patients receiving three cycles demonstrated superior quality‐of‐life (QoL) outcomes at the time of completion of six cycles, reflecting reduced treatment burden. While overall survival (OS) data remain immature, early efficacy analyses have not demonstrated a survival advantage for six cycles over three cycles [4].

Retrospective exploratory data from the Javelin Bladder 100 trial shows the number of cycles of chemotherapy (within the permitted 4–6 range) did not affect outcomes, but there are biases associated with this analysis [3]. Supporting these findings, the Retrospective International Study of Invasive/Advanced Cancer of the Urothelium (RISC), which included 472 patients receiving cisplatin‐ or carboplatin‐based chemotherapy, demonstrated no significant difference in overall survival (OS) between patients who received median four cycles (3–5 cycles) and those who received six cycles (6–9 cycles) (hazard ratio [HR] 1.02, 95% CI: 0.78–1.33, *p* = 0.91) [5]. Similarly, two single‐center retrospective studies from Japan found no significant difference in OS between patients receiving four cycles versus five or more cycles of chemotherapy [6, 7].

These observations are consistent with data from other tumor types, including non‐small‐cell lung cancer, where extending chemotherapy beyond 3–4 cycles failed to improve survival but did lead to increased toxicity [8].

Platinum‐based chemotherapy, particularly cisplatin, is associated with substantial toxicity that may limit the ability to deliver the planned six cycles of treatment. Treatment discontinuation may occur as a result of cumulative toxicity, radiographic disease progression or patient withdrawal [5].

Accordingly, the ability to prospectively identify patients at baseline who are more (or less) likely to complete six cycles of chemotherapy and proceed to maintenance avelumab would be clinically valuable. Early identification of patients unlikely to tolerate or complete prolonged chemotherapy may facilitate more individualized treatment strategies and improve the likelihood of receiving maintenance immunotherapy. Such patients may benefit from shorter durations of induction chemotherapy with an earlier switch to avelumab maintenance or consideration of alternative systemic therapies.

Here, we present a retrospective analysis of patients with mUC treated with platinum‐based chemotherapy, aimed at identifying baseline clinical characteristics and treatment‐related factors associated with successful completion of six cycles of chemotherapy and initiation of maintenance avelumab. We specifically compare cohorts of patients who completed six cycles of chemotherapy without radiographic disease progression with those who discontinued earlier or progressed during chemotherapy in order to identify predictive factors for early chemotherapy cessation.

## 2 Methods

A retrospective cohort analysis was conducted at Barts Cancer Centre, including consecutive patients diagnosed with advanced or mUC between January 2010 and August 2023. 265 patients receiving frontline systemic therapy were identified from electronic patient records. 243 (92%) received frontline platinum‐based chemotherapy with gemcitabine and cisplatin, or gemcitabine and carboplatin. Patients with alternative chemotherapy regimens were excluded. Patients who received chemotherapy as part of peri‐operative treatment (neoadjuvant or adjuvant) and subsequently developed recurrent or metastatic disease within 12 months were also excluded, as they were not given the opportunity to complete the full six cycles of chemotherapy from the outset and therefore were not representative of the study population.

Baseline demographics and clinical characteristics were collected, including age at treatment initiation, sex, Eastern Cooperative Oncology Group (ECOG) performance status, baseline hemoglobin levels, and the presence of liver metastases. Treatment‐related variables including the total number of chemotherapy cycles administered and the occurrence of dose modifications, defined as dose reductions and/or treatment delays, alongside reasons for modifications, were recorded. For patients experiencing dose modifications, the timing, frequency, percentage of dose reduction (expressed as a percentage of the starting dose), and duration of treatment delays were documented.

Completion of chemotherapy was defined as completion of six cycles of frontline platinum‐based chemotherapy without radiographic disease progression, with a disease status assessed according to RECIST v1.1 and confirming stable disease or a response. Patients who discontinued chemotherapy prior to six cycles due to toxicity, radiographic disease progression, or other causes were classified as not completing chemotherapy.

Baseline characteristics of patients who completed the full course of six cycles of chemotherapy without disease progression were compared to those who did not complete six cycles. In addition, patients who experienced chemotherapy dose modifications (dose reductions and/or treatment delays) were compared to those who received chemotherapy without modification. A descriptive analysis was also performed to evaluate patients who experienced a dose reduction during cycles 1–4 and subsequently failed to complete six cycles of chemotherapy without progression.

Statistical analysis was performed using IBM SPSS Statistics v29.0.2.0 (20). All data was coded as categorical variables and Chi‐squared tests were used to compare patient groups.

## 3 Results

Between January 2010 and August 2023, 265 patients receiving frontline systemic therapy for advanced or mUC were identified. Of these, 243 (92%) received platinum‐based chemotherapy with either gemcitabine and cisplatin or gemcitabine and carboplatin. Forty‐nine patients who received chemotherapy as part of a peri‐operative treatment regime (neoadjuvant or adjuvant) were excluded from the analysis as these patients were not intended to receive six cycles of chemotherapy. This resulted in a final cohort of 194 patients eligible for analysis.

Overall, 86 of 194 (44%) patients completed six cycles of frontline platinum‐based chemotherapy without radiographic disease progression. Among patients who completed six cycles, 59% received gemcitabine and cisplatin, 17% had liver metastasis at baseline, 11% had an ECOG performance status (≥ 2), and 6% had low hemoglobin at baseline (Hb < 100 g/L). Baseline demographic and clinical characteristics were broadly similar between those patients who completed six cycles without progression and those who did not (Table 1).

**TABLE 1:** Baseline clinical characteristics of patients who completed six cycles of first‐line chemotherapy without disease progression (PD) versus those who did not.

	Chemotherapy completion 86 (44%)	Did not complete 6 cycles chemotherapy without PD 108 (56%)	*p*
Total patients, 194 (100%)			
Therapy choice			
Gemcitabine/Cisplatin, 101 (52%)	51 (59%)	50 (54%)	0.07
Gemcitabine/Carboplatin, 93 (48%)	35 (41%)	50 (46%)	0.07
Visceral Metastases			
Presence of liver metastases, 39 (20%)	15 (17%)	24 (22%)	0.43
ECOG Performance Status			
PS 0–1, 154 (79%)	72 (84%)	82 (76%)	0.30
PS 2+, 22 (11%)	9 (11%)	13 (12%)	0.30
Hemoglobin (Hb)			
Hb < 100 g/L, 25 (13%)	5 (6%)	20 (19%)	0.13
Dose modifications			
Dose delays, 76 (39%)	30 (35%)	46 (43%)	0.28
Dose reductions, 58 (30%)	12 (14%)	46 (43%)	< 0.001

*Note:* Data are presented as number (percentage) unless otherwise specified. *P* values represent comparisons between avelumab‐eligible and ‐ineligible groups, using chi‐squared tests (SPSS v29).

Abbreviations: ECOG PS, Eastern Cooperative Oncology Group performance status; Hb, hemoglobin.

Dose modifications (defined as dose reductions or treatment delays) were common. Overall, a dose modification was observed in 49% of patients in the chemotherapy completion group compared with 85% of patients who did not complete six cycles without radiographic progression. Experiencing a dose reduction was the only factor significantly associated with failure to complete six cycles of chemotherapy without progression. Dose reductions occurred in 14% of patients who completed six cycles without progression, compared with 43% of patients who did not (*p* < 0.001).

**FIGURE 1:**
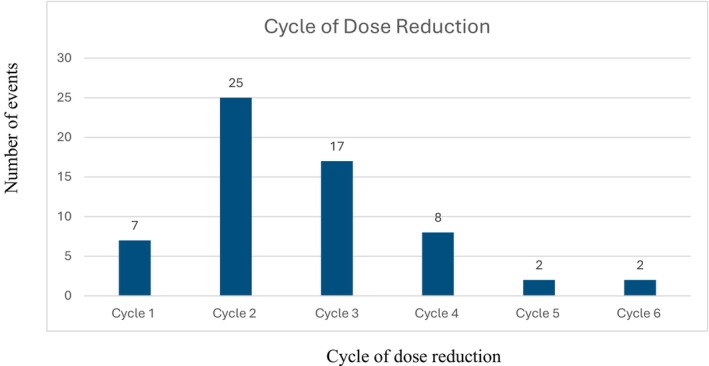
Distribution of chemotherapy dose reductions by cycle during first‐line treatment. The figure illustrates the proportion of patients who required a dose reduction in each cycle, highlighting patterns of early versus later dose modifications.

In total, 61 dose reductions occurred in 58 patients (30%). The majority of dose reductions (*n* = 54) occurred between cycle 1–4, most commonly at cycle 2 (Figure 1). The median time to dose reduction was 21 days (occurring on cycle 2 day 1). The most common dose reduction was to 80% of the starting dose, accounting for 53% of events (*n* = 32). The median overall survival (OS) from the time of dose reduction is 10 months (95% CI: 7.7–11.3). Seven patients experienced more than one dose reduction during their chemotherapy course. Treatment‐related toxicity was the most common reason documented for incurring a dose reduction. The baseline demographics and treatment characteristics of patients who experienced a dose reduction are displayed in Table 2. Patients who experienced dose reductions were more likely to have received gemcitabine and carboplatin, compared to those who did not have a dose reduction (62% vs. 42%, *p* = 0.01). Baseline characteristics, including ECOG performance status, presence of liver metastases, and low baseline hemoglobin levels were similar between patients with and without dose reductions.

**TABLE 2:** Baseline clinical characteristics of patients who experienced versus did not experience dose reductions during first‐line chemotherapy.

	Dose reduction 58 (30%)	No dose reduction 136 (70%)	*p*
Total patients, 194 (100%)			
Therapy choice			
Gemcitabine/Cisplatin, 101 (52%)	22 (38%)	79 (58%)	0.01
Gemcitabine/Carboplatin, 93 (48%)	36 (62%)	57 (42%)	0.01
Visceral Metastases			
Presence of liver metastases, 39 (20%)	12 (21%)	27 (20%)	0.91
ECOG Performance Status			
PS 0–1, 154 (79%)	50 (86%)	104 (75%)	0.13
PS 2+, 24 (12%)	6 (10%)	16 (12%)	0.13
Hemoglobin (Hb)			
Hb < 100 g/L, 25 (13%)	13 (22%)	12 (9%)	0.11

*Note:* Patients receiving gemcitabine/carboplatin were more likely to undergo dose reductions compared with those receiving gemcitabine/cisplatin. Data are presented as number (percentage) unless otherwise specified. *P* values represent comparisons between groups.

Abbreviations: ECOG PS, Eastern Cooperative Oncology Group performance status; Hb, hemoglobin.

Only 19% of patients who underwent a dose reduction within cycles 1–4 subsequently completed six cycles of chemotherapy without radiographic progression. In contrast, 43% of patients who experienced dose reduction within the first four cycles progressed before reaching cycle 6, with a median Progression Free Survival (PFS) of 3 months (95% CI: 2.4–4.1).

**FIGURE 2:**
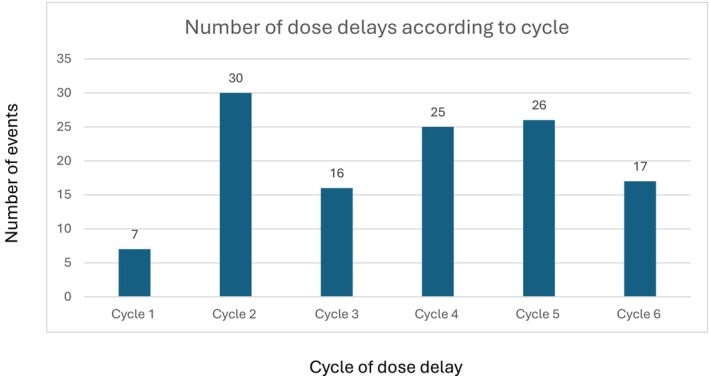
Frequency of chemotherapy dose delays by cycle during first‐line treatment. The figure depicts the number of patients experiencing dose delays in each cycle, showing that delays were more common during the early to mid‐cycles of therapy.

Dose delays were observed in 76 patients (39%), with a total of 121 dose delays recorded. Dose delays most frequently occurred at cycle 2 (Figure 2), with a mean delay of nine days. Patients who experienced a dose delay were more likely to have received frontline gemcitabine and cisplatin, compared with those without dose delays (64% vs. 37%, *p* < 0.001) (Table 3). However, the occurrence of a dose delay at any point during treatment was not associated with completion of six cycles of chemotherapy without disease progression (Table 1).

**TABLE 3:** Baseline clinical characteristics of patients who experienced versus did not experience dose delays during first‐line chemotherapy.

	Dose delays 76 (39%)	No dose delays 118 (61%)	*p*
Total patients, 194 (100%)			
Therapy Choice			
Gemcitabine/Cisplatin, 101 (52%)	27 (36%)	74 (63%)	< 0.001
Gemcitabine/Carboplatin, 93 (48%)	49 (64%)	44 (37%)	< 0.001
Visceral Metastases			
Presence of liver metastases, 39 (51%)	20 (26%)	19 (16%)	0.089
ECOG Performance Status			
PS 0–1, 154 (79%)	61 (80%)	93 (79%)	0.247
PS 2+, 22 (11%)	12 (16%)	10 (8%)	0.247
Hemoglobin (Hb)			
Hb < 100 g/L, 25 (13%)	15 (20%)	10 (8%)	0.526

*Note:* Patients receiving gemcitabine/carboplatin were significantly more likely to have dose delays compared with those receiving gemcitabine/cisplatin. Data are presented as number (percentage) unless otherwise specified. *p* represent comparisons between groups.

Abbreviations: ECOG PS, Eastern Cooperative Oncology Group performance status; Hb, hemoglobin.

## 4 Discussion

It is not clear on the optimal number of cycles of frontline chemotherapy, prior to maintenance avelumab, although the recent DISCUS trial suggests that less than six may be reasonable [4]. The median PFS with frontline platinum‐based chemotherapy ranges from 5.2 to 7.3 months [5, 6, 7, 9], indicating that a substantial number of patients will experience disease progression before completing six cycles of chemotherapy. This is clinically significant in the maintenance era, as patients who progress during chemotherapy are ineligible for maintenance avelumab which is associated with a survival benefit [10]. In our cohort, 43% of patients who underwent a dose reduction within cycles 1–4 progressed before reaching cycle six, with a median PFS of 3.0 months (95% CI: 2.4–4.1), highlighting the risk of attrition during frontline chemotherapy. These findings are in line with data from other studies in mUC and in other tumor types. The aforementioned retrospective data in frontline mUC from Japan indicates progressive disease rates of 18.4%, 19.2%, and 30.6%, within the first two cycles, between cycles 2–4, and between cycles 4–6, respectively [6], further illustrating the challenge of delivering six cycles of chemotherapy in routine practice. Similar observations have been reported in other tumor types. In a randomized trial comparing three versus six cycles of mitomycin/vinblastine/cisplatin (MVP) chemotherapy in advanced lung cancer, only 31% of patients randomized completed all six cycles [11]. Treatment discontinuation was primarily attributed to toxicity, progression, or patient preference. No significant survival benefit was observed with six cycles compared to three cycles, suggesting diminishing returns with prolonged chemotherapy exposure [11].

It would therefore be helpful to predict from baseline characteristics which patients are likely to successfully complete six cycles of chemotherapy and identify patients who may benefit from an earlier switch to immunotherapy. Previous data in other tumor types has shown conflicting effects of prognostic factors such as poor performance status at baseline. One randomized study in advanced lung cancer demonstrates no difference in survival benefit between patients with poor performance status and duration of chemotherapy [11], whilst another found that patients with worse performance status seemed to benefit from prolonged chemotherapy [12]. In our analysis, baseline demographic and clinical characteristics (including liver metastases, low hemoglobin at baseline, and ECOG performance status ≥ 2) were not associated with the ability to complete six cycles of chemotherapy without progression. Our data identifies challenges in predicting which patients will successfully complete the full six cycles of chemotherapy in advanced or mUC at baseline, suggesting that baseline prognostic factors alone are insufficient to reliably identify patients at risk of early treatment discontinuation.

These discrepancies highlight the complexity of predicting outcomes based on baseline characteristics. It is therefore reasonable to set out with the intention that all patients will be offered the full six cycles at the point of treatment initiation.

However, our findings indicate that the requirement for a dose reduction between cycles 1–4 is the most important predictor of inability to complete six cycles of chemotherapy without progression. Notably, only 19% of patients who underwent an early dose reduction ultimately completed six cycles, and 81% discontinued chemotherapy prematurely. This observation suggests that treatment‐related toxicity leading to dose reduction may represent an early marker of early cessation of treatment and risk of attrition prior to maintenance therapy. In this context, early dose reduction may identify a subgroup of patients in whom continued chemotherapy escalation confers limited benefit and may compromise the opportunity to receive maintenance avelumab.

Dose delays, however, were not associated with failure to complete six cycles without progression. The lack of association between dose delays and chemotherapy completion may reflect the heterogeneous causes of treatment delays, including logistical factors, transient toxicity, or patient preference, which may not necessarily indicate reduced chemotherapy tolerance.

The study has several limitations. First, the data obtained is retrospective in nature from a single center and lacks randomization, which may limit generalizability. There was heterogeneity in chemotherapy regimens with some patients receiving carboplatin versus cisplatin, which may influence toxicity profiles, likelihood of dose modifications, and patient outcomes. Furthermore, there are currently no standardized guidelines for dose modifications such as dose reductions or delays, or early cessation of treatment. Additionally, we did not perform multivariable analyses to adjust for potential confounding factors. This was due to the limited number of events and the strong correlation between treatment‐related variables, which constrained the robustness and interpretability of adjusted models. As a result, the associations identified should be interpreted as exploratory and hypothesis‐generating rather than causal.

These limitations highlight the need for more rigorous, multicentre, prospective studies to clarify the optimal chemotherapy duration and to define clear guidelines for dose modifications.

## 5 Conclusion

Whilst the treatment landscape for mUC has changed with the introduction of EVP as a new standard of care [1, 2], it is not yet universally available, and many patients are still being offered platinum‐based chemotherapy as frontline treatment. The rationale for giving 6 cycles of chemotherapy in mUC is unclear [3, 5, 6, 7, 8]. It is likely some patients will benefit from shorter periods of chemotherapy, as is the case in lung cancer [8]. Knowing which patients should stop early is useful as one can plan to sequence immune therapy, especially after recent data suggests similar outcomes and better quality of life with only three cycles [4]. Our data suggest the duration of chemotherapy cannot be adequately predicted from baseline characteristics and pre‐planned shorter chemotherapy regimens are questionable. It is reasonable that all patients should be offered the full six cycles. However, if difficulties administering treatment are encountered, particularly initiating dose reductions, considering shorter periods of chemotherapy (3–4 cycles) may be useful, to give patients the opportunity to receive maintenance avelumab. There is value to conducting randomized control trials to assess shorter periods of chemotherapy.

## Author Contributions

**Francesca Jackson‐Spence:** conceptualization, investigation, writing – original draft, methodology, writing – review and editing, data curation, software, formal analysis, project administration, visualization. **Domiziana Aspden:** data curation. **Sofia Diaz:** data curation. **Vishwani Chauhan:** data curation. **Catherine Graham:** data curation. Matthew young: writing – review and editing. **John Connor Wells:** writing – review and editing. **Sara Coca Membribes:** writing – review and editing. **Charlotte Ackerman:** project administration, writing – review and editing. **Bernadett Szabados:** writing – review and editing. **Thomas Powles:** conceptualisation, investigation, writing – original draft, methodology, writing – review and editing, data curation, project administration, visualization.

## Funding

The authors have nothing to report.

## Ethics Statement

Ethical approval for this study was not required.

## Consent

A retrospective institutional audit on treatment‐naive patients with metastatic urothelial cancer was performed on patients treated at St Bartholomew's Hospital, Barts Health NHS Trust, London. This work was performed within the context of a service improvement project and registered with the local institutional board (ID: 13609, Title: Real World Outcomes with Systemic Therapy for Kidney and Bladder Cancers at St Bartholomew's Hospital); therefore, no informed consent was sought from patients.

## Conflicts of Interest

F. Jackson‐Spence has received travel expenses from EUSA and Merck and has received honoraria from MSD. T. Powles has received travel expenses and research funding from Roche, Pfizer, MSD, AstraZeneca, Ipsen, BMS, Merck, Exelixis, Novartis, Seattle Genetics, Merck Serono, Astellas, Johnson & Johnson, and Eisai, and has received honoraria from Roche, Pfizer, MSD, AstraZeneca, Ipsen, BMS, Merck, Exelixis, Incyte, Novartis, Seattle Genetics, Merck Serono, Astellas, Johnson & Johnson, and Eisai. B. Szabados has received travel expenses and research funding from Roche, Genentech, Merck Sharp & Dohme, Pfizer, and Bristol Myers Squibb, and has received honoraria from Merck, Roche, Pfizer, Ellipses, and Ipsen. M. Young has received travel expenses from Pfizer and Ipsen, and has received horararia from Pfizer, Merck, Eisai, and Ipsen. He has also received a consultancy/advisory fee for Novartis, EMD Serono, Ipsen, and Pfizer. S Membribes has travel expenses from BMS, Roche, and Merck. The authors have no other relevant affiliations or financial involvement with any organization or entity with a financial interest in or financial conflict with the subject matter or materials discussed in the manuscript apart from those disclosed.

## Data Availability

The data that support the findings of this study are available from the corresponding author upon reasonable request.
